# Unique SARS-CoV-2 Variants, Tourism Metrics, and B.1.2 Emergence in Early COVID-19 Pandemic: A Correlation Analysis in South Dakota

**DOI:** 10.3390/ijerph20186748

**Published:** 2023-09-13

**Authors:** Ahmed Nahian, Victor C. Huber, Lisa M. McFadden

**Affiliations:** Division of Basic Biomedical Sciences, University of South Dakota, 414 E. Clark St., Vermillion, SD 57069, USA

**Keywords:** SARS-CoV-2, tourism, B.1.2, genomics

## Abstract

The severe acute respiratory syndrome coronavirus-2 (SARS-CoV-2) virus, which is the source of the coronavirus disease 2019 (COVID-19), was declared a pandemic in the March of 2020. Travel and tourism were severely impacted as restrictions were imposed to help slow the disease spread, but some states took alternative approaches to travel restrictions. This study investigated the spread of COVID-19 in South Dakota during the early pandemic period to better understand how tourism affected the movement of the virus within the region. Sequences from the fall of 2020 were retrieved from public sources. CDC and other sources were used to determine infections, deaths, and tourism metrics during this time. The data were analyzed using correlation and logistic regression. This study found that the number of unique variants per month was positively correlated with hotel occupancy, but not with the number of cases or deaths. Interestingly, the emergence of the B.1.2 variant in South Dakota was positively correlated with increased case numbers and deaths. Data show that states with a shelter-in-place order were associated with a slower emergence of the B.1.2 variant compared to states without such an order, including South Dakota. Findings suggest complex relationships between tourism, SARS-CoV-2 infections, and mitigation strategies. The unique approach that South Dakota adopted provided insights into the spread of the disease in areas without state-wide restrictions. Our results suggest both positive and negative aspects of this approach. Finally, our data highlight the need for future surveillance efforts, including efforts focused on identifying variants with known increased transmission potential to produce effective population health management.

## 1. Introduction

The coronavirus disease 2019 (COVID-19), caused by the severe acute respiratory syndrome coronavirus-2 (SARS-CoV-2) virus, began spreading in 2019 and quickly became a pandemic in March 2020. SARS-CoV-2 spreads through contact with aerosols from an infected person [1]. To reduce the chance of exposure to this disease, many places encouraged people to stay at home. This meant that the travel and tourism industries were among the most severely impacted sectors as the world dealt with this unprecedented global medical, societal, and financial emergency. International visitor arrivals decreased by 74% between 2019 and 2020, and revenue from international travel is anticipated to have decreased by 11-fold, the largest decrease since the end of the global financial crisis of 2009 [2]. According to the US Census, American employees in the travel, tourism, and outdoor recreation industries saw large pay declines during the second quarter (up to 40%) in 2020 compared to 2019 [3].

In addition, daily travel statistics from the U.S. Bureau of Transportation Statistics show that daily trip counts significantly decreased in 2020 [4]. The adjusted travel trend in 2019 and 2020 were lower than it was prior to the pandemic, and this pattern showed that staying at home more frequently, and traveling less, may have become a norm in American culture compared to the years preceding the pandemic. These trends had a significant impact on states where tourism is high. With only 880,000 permanent residents, South Dakota hosts approximately 12–14 million visitors annually [5,6], with a significant increase in tourism during the summer. South Dakota was one of seven states that did not impose a statewide shelter-in-place order in the initial wave of COVID-19, and they declared that the state would “return back to normal” on April 28, 2020 [7,8]. Thus, South Dakota became one of the first states to openly welcome tourism during the first year of the pandemic. In 2020, South Dakota saw only a modest decline (18%) in visitor expenditures, and only a 13% decline in visitors when compared to 2019 [5]. This trend quickly reversed in the subsequent years. Given that most states encouraged a reduction in tourism and travel to slow down the spread of SARS-CoV-2, South Dakota took a unique approach to travel and tourism that allows for rare insights into the impact of tourism on population health.

As it still continues to infect people, SARS-CoV-2 has shown an ability to rapidly mutate and evolve from the classical variant (Wuhan-type) virus that was first isolated. These mutations are thought to influence infection risk and control strategies [9]. From December 2019 to December 2020, 15 clades of SARS-CoV-2 were detected [10]. By comparison, only ten clades emerged in 2021, suggesting that 2020 was a period of rapid change in SARS-CoV-2. The product of these periods of rapid change was the emergence of new variants. The suspected time of emergence of the most recent common ancestors of the Alpha, Beta, Gamma, and Delta variants occurred late in 2020. These variants were all associated with higher transmissibility, and some were associated with greater clinical disease severity [10]. Indeed, the United States saw one of the deadliest waves of SARS-CoV-2 infections occurring in late 2020 and early 2021 [11]. Understanding how different virus mutations spread through communities is important for analyzing the impact of these mutations on population health and healthcare management.

The transitory movement of people into South Dakota due to tourism allows for the seeding of the variants of SARS-CoV-2 in the state, with the potential for significant and unexpected impacts on healthcare infrastructure. While many states implemented shelter-in-place orders during 2020, South Dakota’s alternative approach creates a unique opportunity to investigate the spread of this disease. The goal of the current study was to examine the emergence of different mutations of SARS-CoV-2, measured using variants, that occurred in South Dakota during the summer and fall of 2020. Specifically, we hypothesized that tourism leads to the seeding of new variants of the virus in the state. Second, we examined the impact of these new variants on cases and deaths in the state. Finally, to better understand the impact of statewide efforts to reduce travel, we compared the emergence of a specific variant, B.1.2, in states with and without a shelter-in-place order.

## 2. Materials and Methods

### 2.1. Ethical Statement

All studies were approved by the University of South Dakota (USD) Institutional Review Board. Collection dates and locations more specific than state and year were deemed to be Protected Health Information and therefore were not made publicly available through NCBI Virus for samples collected using USD. Metrics were binned according to month to ensure that patients were not identifiable.

### 2.2. Metrics

Unique variants: All SARS-CoV-2 variant sequences from South Dakota between 1 May 2020 and 30 November 2020 were downloaded from NCBI Virus on 2 November 2022, resulting in 813 sequences (see Supplementary Materials for accession numbers). Collection dates were then associated with samples, and the number of unique variants per month was calculated in Tableau (22.2.1; Seattle, WA, USA). The proportion or percentage of sequences assigned to B.1.2 was also calculated month-wise.

SARS-CoV-2 cases and deaths: Clinical cases and deaths in the state of South Dakota from 1 May 2020 to 30 November 2020 were downloaded from CDC (data.cdc.gov; accessed on 25 May 2023). Cases and deaths were then binned according to month.

Tourism: Tourism measures were obtained from the South Dakota Tourism Industry’s Research Reports website [12]. Total visitations and hotel occupancy from May to November 2020 were recorded.

B.1.2 in shelter vs. non-shelter states: Moreland et al. and Kates et al.’s methods were used to determine the states that did not enact a shelter-in-place order [7,8]. South Dakota, Iowa, and Utah were listed as states with no shelter-in-place orders in both sources. These states were then matched according to population size with states that did enforce a shelter-in-place order, resulting in the selection of Delaware, Nevada, and Connecticut for comparison. All sequences between 1 May 2020 and 30 November 2020 were downloaded from GISAID [13] for these states (5334 sequences), given that a greater number of samples were available in GISAID compared to NCBI Virus. The percentage of B.1.2-positive sequences month-wise was calculated for each state.

### 2.3. Analysis

Non-linear increases in SARS-CoV-2 cases and deaths were observed during this time period; therefore, non-parametric analysis was used. Spearman’s Rho Correlation Coefficients were calculated. Bivariate correlations among clinical metrics (cases and deaths), variants (monthly unique variants and proportion of B.1.2), and tourism metrics (total visitations and hotel occupancy) were calculated in SAS Studio. Logistic regression was used to compare B.1.2-positive samples over time and shelter order. Briefly, the Logistic Procedure was used. The outcome variable (B.1.2 or all other lineages) was predicted using Shelter-Order (Yes or No) and Month (May, June, July, August, September, October, and November), as well as the model intercept. For more information on logistic regression using Proc Logistic, please see [14]. Significance was set at *p* < 0.05.

## 3. Results

### 3.1. SARS-CoV-2 Variants

**Figure 1:**
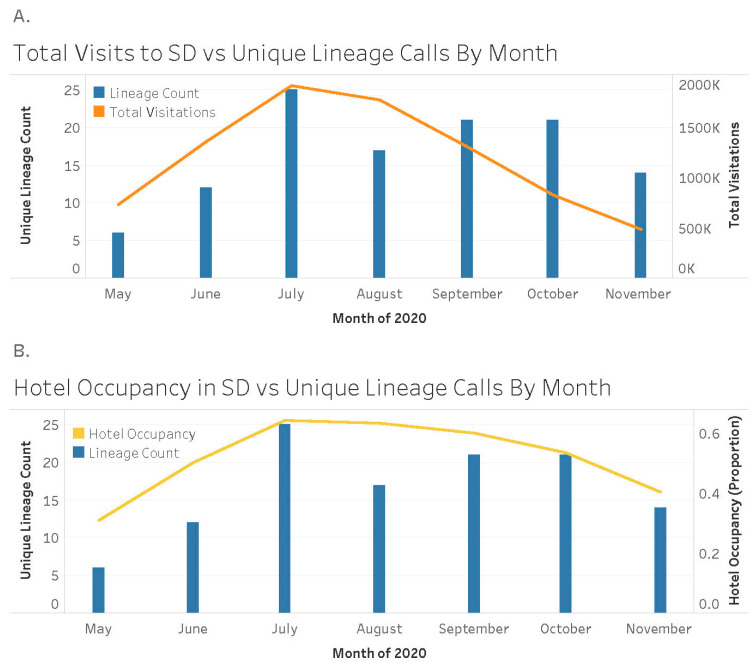
Associations between variant counts and tourism. All samples from South Dakota were downloaded from NCBI Virus for the period of May 2020 to November 2020. The number of unique variants per month was calculated and compared to total visits to the state (**A**) and hotel occupancy (**B**) during this time. Hotel occupancy was significantly correlated with unique SARS-CoV-2 variants detected per month.

The number of unique SARS-CoV-2 variants per month was compared with measures of tourism: total visitations to the state and hotel occupancy. While unique variants detected on a per-month basis were non-significantly correlated with total visits to South Dakota (r = 0.52, *p* = 0.22; Figure 1), there was a significant positive correlation with hotel occupancy (r = 0.85, *p* = 0.02; Figure 1). However, the number of unique variants per month did not significantly correlate with either cases per month (r = 0.16, *p* = 0.72) or deaths per month (r = 0.16, *p* = 0.72).

### 3.2. Clinical Metrics

**Figure 2:**
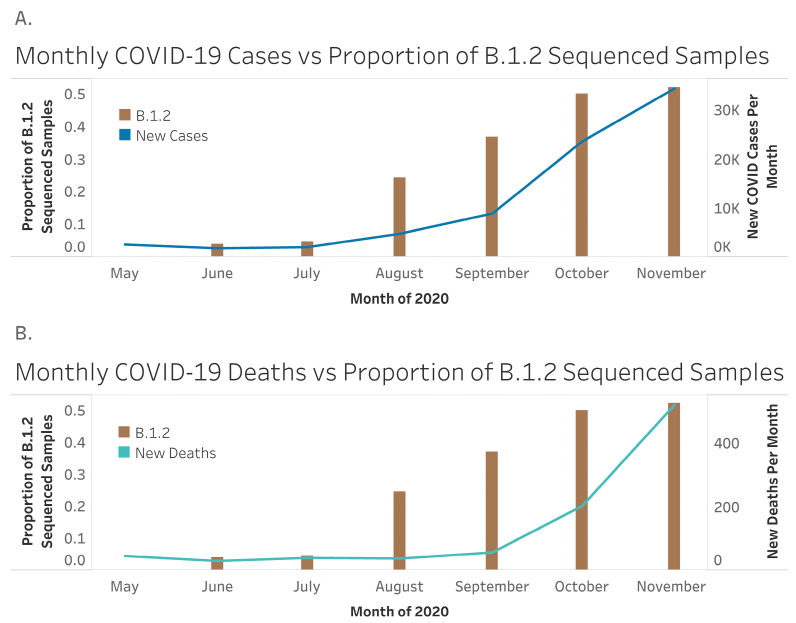
Associations between B.1.2, infections and deaths. All samples from South Dakota were downloaded from NCBI Virus for the period of May 2020 to November 2020. The proportion of the sequences from the B.1.2 lineage was calculated per month, and these numbers were compared with the number of new COVID-19 cases per month in the state (**A**), and the number of new COVID-19 deaths in the state (**B**) during this time. Cases and deaths were significantly correlated with the proportion of B.1.2-positive samples per month.

Monthly cases (r = −0.64, *p* = 0.12) and deaths (r = −0.75, *p* = 0.05) due to COVID-19 were negatively correlated with total visitations to South Dakota. Hotel occupancy was also negatively correlated with monthly deaths (r = −0.32, *p* = 0.48), and weakly correlated with monthly cases (r = −0.18, *p* = 0.70). Interestingly, COVID-19 cases and deaths in the state of South Dakota increased as B.1.2 spread. As B.1.2 made up an increasing proportion of variants represented in the samples sequenced in a given month, new cases increased (r = 0.89, *p* = 0.01; Figure 2). Deaths also increased as the proportion of B.1.2 lineage samples increased (r = 0.75, *p* = 0.05; Figure 2).

#### In Shelter vs. Non-Shelter-In-Place States

**Figure 3:**
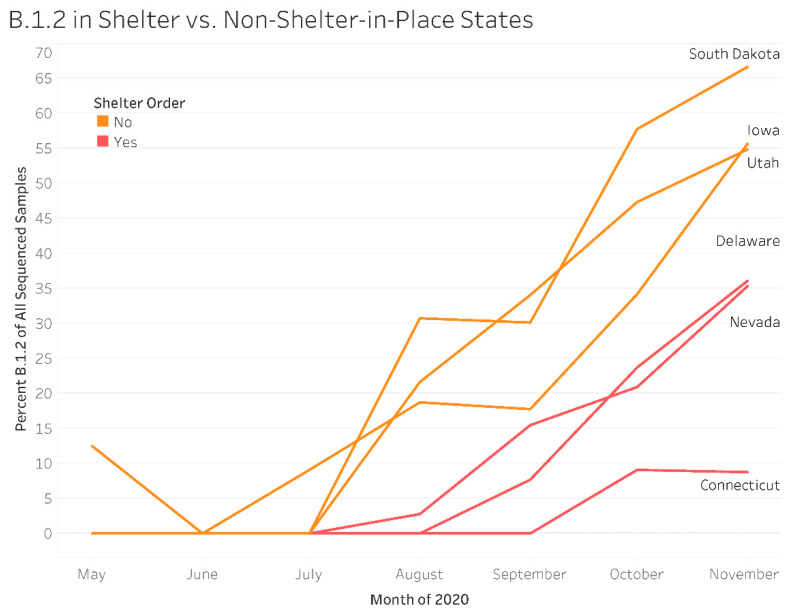
The emergence of the B.1.2 lineage in states with and without shelter-in-place orders. All sequences collected from May 2020 to November 2020 in the Global Initiative on Sharing All Influenza Data (GISAID) database from Iowa, South Dakota, Nevada, Utah, Delaware, and Connecticut were downloaded. The percentage of B.1.2-positive sequences was calculated by month in states with (Delaware, Nevada, and Connecticut) and without (South Dakota, Iowa, and Utah) shelter-in-place orders. States with shelter-in-place orders had lower odds of having B.1.2 lineage-positive sequences.

Given that B.1.2 was highly correlated with COVID-19 clinical metrics, the role of travel restrictions (shelter-in-place orders) was further investigated. There was a significant effect of time (χ(6) = 455.05, *p* < 0.05) and shelter-in-place order (χ(1) = 136.31, *p* < 0.05). As expected, all months prior to November 2020 (reference group) had significantly lower odds of a sequenced sample being assigned a B.1.2 variant designation (Table 1). The odds of a sequenced sample being assigned to the B.1.2 variant were 3.57 times higher in states where no shelter-in-place orders were issued compared to their population-matched shelter-in-place states. States, where no shelter-in-place order was issued, saw an earlier emergence of this lineage, leading to higher proportions throughout the fall when compared to shelter-in-place states (Figure 3).

## 4. Discussion

The current study explored the spread of SARS-CoV-2 variants through South Dakota in the early stages of the pandemic (May 2020–November 2020). Since South Dakota took a unique approach to public health mitigation measures, this study investigated the spread of SARS-CoV-2 in the state during a time when other states were more severely limiting travel. Our results show that the number of unique SARS-CoV-2 variants in a month was positively correlated with a major tourism indicator, hotel occupancy. Surprisingly, increases in the number of variants did not positively correlate with the number of cases or deaths in South Dakota. Instead, our data show that the arrival of a specific SARS-CoV-2 variant, B.1.2, positively correlated with the increased case numbers and deaths. Although it did appear that tourism increased the diversity of the virus present in the state, the number of cases and deaths seemed to be influenced by the presence of a specific variant during the time period included. Finally, state shelter-in-place mandates were associated with a slower emergence of the B.1.2 lineage when compared to those states that did not have such restrictions in place. Together, these findings highlight the complex relationships between tourism, SARS-CoV-2 infections, mitigation strategies, and public health.

Prior research suggests that mobility impacts the spread of SARS-CoV-2. Studies have demonstrated that restricting mobility can lessen the burden on healthcare services and aid in slowing down the spread of infection. According to Jia et al., the COVID-19 transmission rate significantly decreased in Wuhan, China, when social distance measures were put in place [15]. Similar findings were obtained by Kuchler et al., who reported that stay-at-home orders issued in the US decreased mobility and slowed the virus’s transmission [16]. Mobility data were used to predict the spread of COVID-19 in a study by Badr et al., with these researchers suggesting a methodology that makes use of such data to estimate the number of cases [17]. Kraemer et al. discovered that early actions that limited mobility, like social exclusion and school closings, were successful in slowing down the spread of the virus [18]. According to Fong et al., travel restrictions implemented by many nations during the pandemic were successful in limiting SARS-CoV-2 transmission, but their efficacy relied on the timing and severity of the restrictions [19]. In a study conducted in Italy, Colombo et al. found that movement patterns had a substantial influence on the spread of SARS-CoV-2, and suggested using mobility data to guide public health policy [20]. In a study carried out in the United States by Chinazzi et al., it was discovered that social isolation policies, school closures, and mobility restrictions all reduced the spread of SARS-CoV-2 [21]. Bi et al. found that rigorous mobility limitations, such as travel bans and quarantines, helped to slow down the transmission of the virus by analyzing data from Wuhan, China [22]. In Tokyo, Japan, Arima et al. looked at the connection between mobility patterns and the spread of SARS-CoV-2, ultimately reporting that less mobility during rush hours was linked to fewer COVID-19 cases [23]. Generally speaking, these studies highlight the significance of efficient public health measures in containing the spread of the COVID-19 pandemic. These findings show that mobility patterns play a significant role in the spread of SARS-CoV-2, and that interventions that limit mobility, such as social isolation policies, stay-at-home directives, and travel restrictions, can be successful in reducing the spread of this virus.

Prior research also suggests an association between tourism and SARS-CoV-2 infection. For example, when modeling metro areas in South Carolina, Charleston’s transmission rate was two times higher than other metros in the state [24]. Findings suggested that transportation and tourism activities in the Charleston area influenced this higher rate of transmission. Wastewater surveillance of SARS-CoV-2 in Las Vegas also suggested that tourism plays an important role in the abundance of the virus’ RNA [25]. Specifically, visitors were estimated to contribute more than 60% of the viral RNA load to the sewershed serving the Las Vegas Strip. In a coastal community in Florida, wastewater results suggested that SARS-CoV-2 RNA increased by 1.06 Log10 genomic copies/L per 100 tourists weekly [26]. Tourism was also associated with hospital encounters and healthcare capacity. Seasonal variations were important predictors of tourist visits to the emergency department compared to resident patients [27]. Further, the authors noted that non-metropolitan areas with limited healthcare capacity were especially impacted by tourist emergency department presentations of COVID-19. Similar findings were observed in Japan following travel campaigns [28]. Specifically, the authors noted that the travel campaign may have influenced the spread of the disease from urban centers to nonurban locations where healthcare capacity is limited. In contrast, a study of German residents early in the pandemic did not find tourism to be related to infection rates [29]. Finally, in an analysis of 91 countries, tourism was a top five determinant of SARS-CoV-2 morbidity and mortality, especially in early 2020, when travel was more likely to be restricted [30]. These prior studies further suggest an association between tourism and the spread of COVID-19.

Prior publications have also investigated the spread of COVID-19 from events in South Dakota during the timeframe of the current study. The cases studied by the Minnesota Department of Health centered around a motorcycle rally hosted in South Dakota from August 2020 to September 2020 [31]. The study showed a relatively small number of cases (86 cases) that were associated with this mass-gathering event of ~460,000 people. A second study that focused on attendees of the same event reported 649 COVID-19 cases across the US, which remains relatively small when considering the size of the event [32]. Thus, the ability to track and truly evaluate the impact of virus transmission, even in a population that very likely does not have pre-existing immunity against this virus, is very difficult. Limitations of both studies included an underrepresented population of potentially infected individuals and an inability to properly identify SARS-CoV-2 variants that were represented in the population. These studies not only highlight the potential of events and tourism to spread the disease, but also highlight the challenges of linking cases in the population to specific events.

While limiting travel and social interactions can help to prevent the spread of SARS-CoV-2, it is clear that these policies also had a significant impact on mental and physical health. Numerous studies have shown that stay-at-home policies were associated with increased depression, anxiety, and other mental health concerns, especially in people with pre-existing mental health disorders and the elderly [33,34,35,36]. Excessive deaths from other diseases, such as cardiac arrests, liver disease, and diabetes, were also noted during the pandemic [37,38,39]. Similarly, excessive deaths, especially in younger adults, due to accidents, motor vehicle accidents, drug overdoses, assaults, and homicides were observed [40]. It should be noted that the role of shelter-in-place orders is less clear in these excessive deaths. While travel restrictions helped to slow down the spread of this disease, there were likely unintended consequences for physical and mental health. This further complicates a true estimation of the burden of the COVID-19 pandemic on the healthcare system, as there were multiple health conditions that were exacerbated during this time.

As a potential intervention for limiting healthcare burden, the COVID-19 pandemic showed us that balancing the need to reduce the spread of SARS-CoV-2 through stay-at-home directives and promoting mental and physical health is difficult. Findings of the current study suggest that hotel occupancy was associated with increased introduction of SARS-CoV-2 variants into South Dakota but not an increase in infections or mortality. Rather, specific variants, such as B.1.2, which had adventitious mutations that helped to increase transmissibility, were also associated with the increase in infections and deaths. Restricting mobility and tourism through stay-at-home orders may be most effective when the known impending spread of virus variants with significantly higher infection or mortality rates is imminent [41,42]. When less-transmissible variants are circulating, allowing normal activities to occur may help to mitigate excessive deaths due to other causes. It is crucial to further study the complexity and nuanced aspects of this issue in regard to the transmission of SARS-CoV-2 and the effects of stay-at-home policy on mental and physical health. A more thorough examination of the impact of public health policies on physical and mental health is needed.

Examining unique mutations observed in SARS-CoV-2 variants that were associated with increased virus spread and deaths may also provide clues for developing effective warnings for new outbreaks. In the current study, detection of the B.1.2 variant was associated with periods of higher infections and mortality. The United States experienced a rise in the percentage of B.1.2-positive sequences starting in June 2020 [43,44]. Percentages peaked around 1 December 2020, with B.1.2 accounting for approximately 50% of sequenced samples in the United States. Since late 2019, SARS-CoV-2 has undergone a number of modifications that could affect the transmission of SARS-CoV-2. Early in the pandemic, the spike protein incorporated the D614G mutation, which likely contributed to the virus being more contagious [45]. This mutation swiftly took over, and it is now present in almost all SARS-CoV-2 isolates in circulation. The B.1.2 lineage had multiple mutations, including the D614G spike protein signature. The B.1.2 variant was noted to have a moderate transmission fitness advantage over B.1, especially in the Midwest [46,47,48], and this advantage was modest compared to emerging lineages such as B.1.1.7, B.1.526, B.1.427, and B.1.429. However, it was noted that B.1.2 persisted to dominate in circulation even when new variants emerged [46]. Kepler et al. noted that the spread of B.1.2 may also have been facilitated by spatial transmission advantages, particularly in the Midwest. Indeed, findings from the current study support this position [46]. The B.1.2 variant emerged early, and increased to a greater percentage of samples sequenced in states where shelter-in-place orders were not issued compared to size-matched states where these measures were in place. Moreover, these findings highlight the complex interaction of viral genetics and human behavior in the spread of SARS-CoV-2. While there are still many unknown factors that facilitated the spread of B.1.2, this SARC-CoV-2 variant was associated with the increase in infections and deaths in the state of South Dakota.

### Limitations

The current study is not without limitations, but there is also a lot to learn from studies like this that make early observations during the circulation of a novel pandemic virus. For one, the time period included was chosen to reduce other factors that may influence the spread of SARS-CoV-2, including the potential that prior immunity and/or re-infection with SARS-CoV-2 will be minimized. Similarly, since vaccines against SARS-CoV-2 were not available until December 2020, vaccine-induced immunity does not have to be considered as a potential factor limiting virus infection during this time. Future studies will focus on similar evaluations of SARS-CoV-2 variants when prior immunity against SARS-CoV-2 will have to be considered as a factor in virus spread. Another potential limitation of the results presented here includes sampling biases of the sequenced SARS-CoV-2 samples, which could impact our knowledge of viruses that were detected in South Dakota. Next, the approach taken to mitigate the spread of disease by state officials was unique in comparison to much of the country. Additionally, South Dakota is a small, rural state, and states with greater population densities than those found in South Dakota may lead to different outcomes. Thus, these results may not be generalizable to other populations. Finally, correlative relationships, and not causal relationships, were explored. Given the uniqueness of the data, causal outcomes should not be inferred.

## 5. Conclusions

Tourism metrics were associated with the increase in the unique variants of SARS-CoV-2, but not clinical metrics. Instead, the spread of a specific variant, B.1.2, was associated with the increase in cases and deaths. The findings from the current study suggest that the increase in the number of unique variants in the state is not as clinically relevant as identifying a variant that is known to increase hospitalizations and deaths. This implies that the number of unique variants that spread both into and within the state is not as important for cases and deaths as the expansion of one lineage upon arrival. Using this knowledge, we can move future surveillance efforts forward by looking for the arrival of variants that are known to readily transmit among the population. Specifically, we can now focus on surveillance directed toward identifying a specific variant, and making informed healthcare decisions that can improve population health management.

## Figures and Tables

**Table 1:** Logistic regression of B.1.2 emergence.

Measure	Level	Odds Ratio	95% Confidence Interval
Shelter-in-Place Order			
	No	3.572	(2.885, 4.423)
	Yes	Reference	
Month			
	May	<0.001	(<0.001, 0.004)
	June	0.021	(0.012, 0.036)
	July	0.159	(0.118, 0.214)
	August	0.199	(0.161, 0.246)
	September	0.304	(0.224, 0.412)
	October	0.670	(0.540, 0.855)
	November	Reference	

## Data Availability

Publicly available datasets were analyzed in this study. Those data can be found here: https://www.ncbi.nlm.nih.gov/labs/virus/vssi/#/, see supplemental information for accession numbers (accessed on 2 August 2023); https://gisaid.org, EPI_SET ID: EPI_SET_230802qz (accessed on 2 August 2023).

## Supplementary Materials

The following are available online at https://www.mdpi.com/article/10.3390/ijerph20186748/s1, Accession numbers for NCBI sequences.
